# Supplementary material for: Isomadecassoside, a New Ursane-Type Triterpene Glycoside from Centella asiatica Leaves, Reduces Nitrite Levels in LPS-Stimulated Macrophages
Source: Biomolecules. 2021 Mar 25;11(4):494. doi: 10.3390/biom11040494 (PMC8064468; doi:10.3390/biom11040494)

Supplementary Materials

Isomadecassoside, a New Ursane-Type Triterpene Glycoside from Centella asiatica Leaves, Reduces Nitrite Levels in LPS-Stimulated Macrophages

Giuseppina Chianese[1], Francesca Masi[1], Donatella Cicia[1], Daniele Ciceri[2], Sabrina Arpini[2], Mario Falzoni[2], Ester Pagano[1,3], Orazio Taglialatela-Scafati[1,*]

Supporting data **Page**

S1: ^{1}H NMR spectrum of isomadecassoside 2
S2: 2D NMR HSQC spectrum of isomadecassoside 2
S3: 2D NMR HMBC spectrum of isomadecassoside 3
S4: 2D NMR COSY spectrum of isomadecassoside 3
S5: 2D NMR NOESY spectrum of isomadecassoside 4

Figure S1: ^1H NMR spectrum of isomadecassoside (CD_3OD, 700MHz)

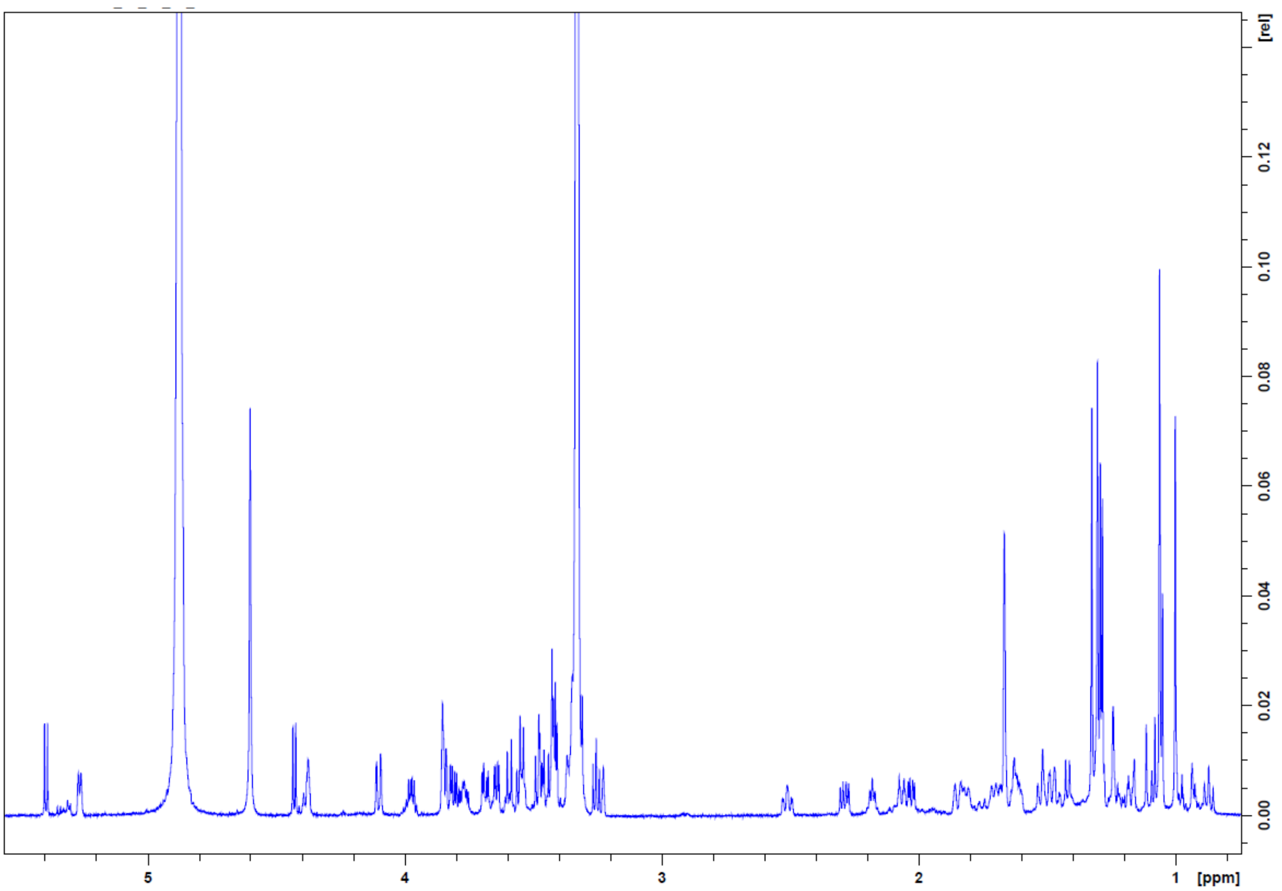

Figure S2: 2D NMR HSQC spectrum of isomadecassoside (CD_3OD)

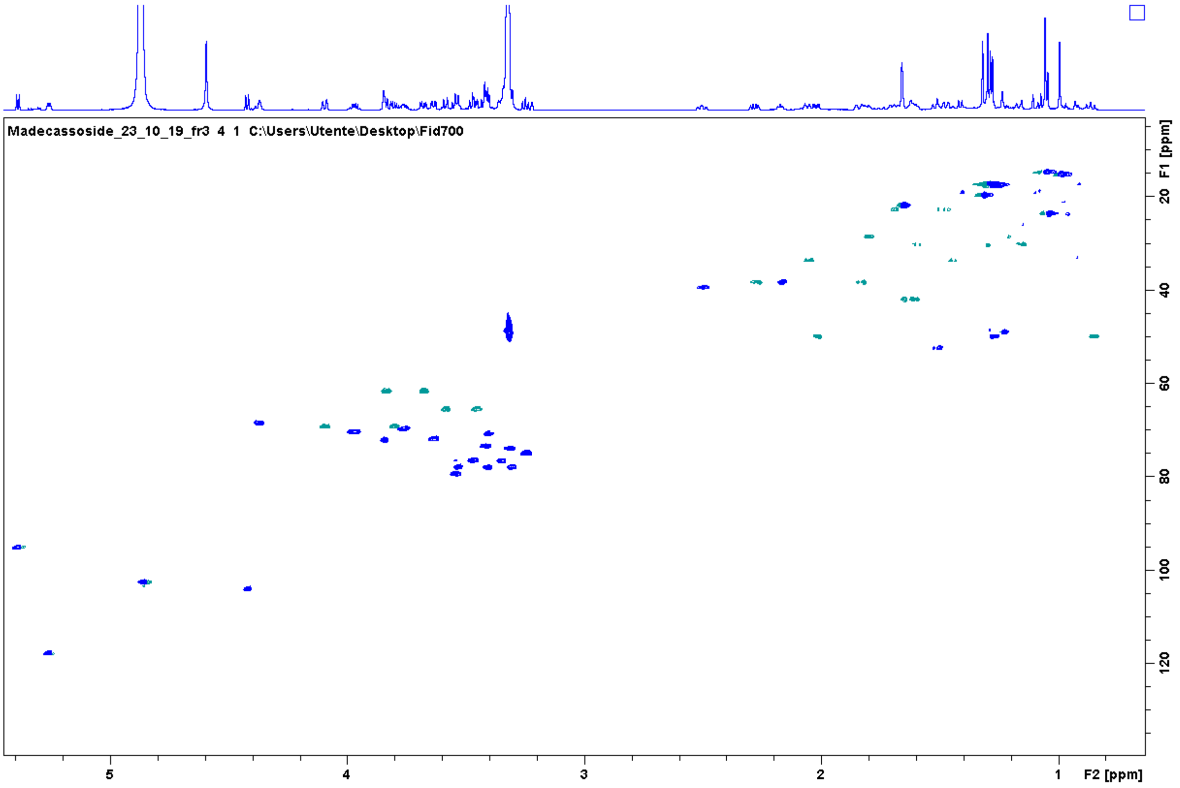

Figure S3: 2D NMR HMBC spectrum of isomadecassoside (CD_3OD)

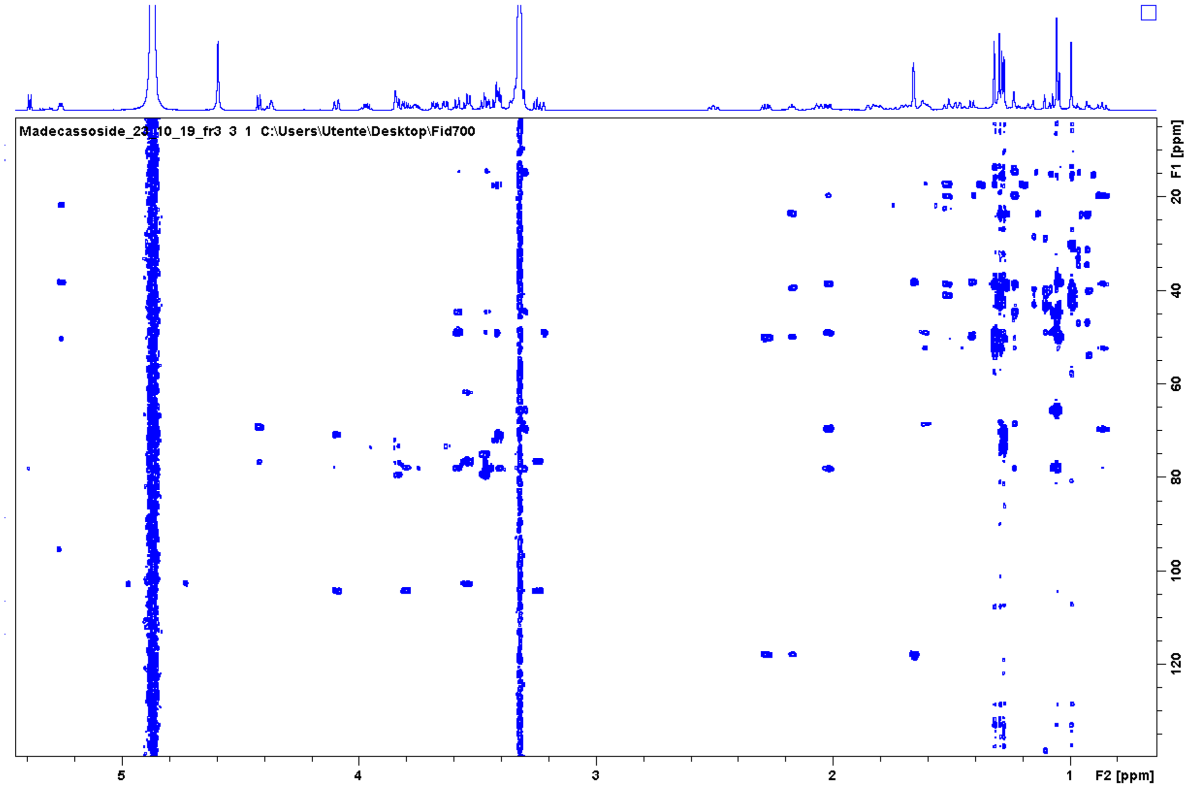

Figure S4: 2D NMR COSY spectrum of isomadecassoside (CD_3OD)

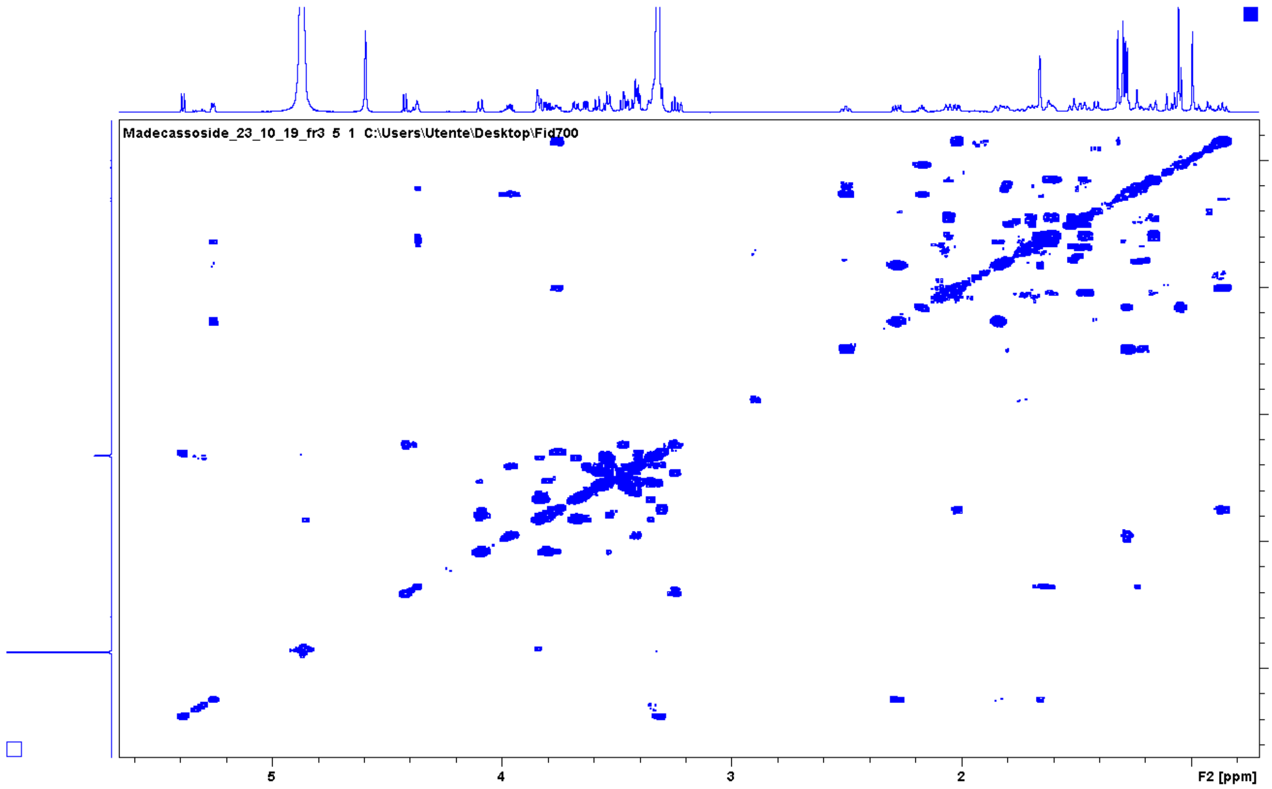

Figure S5: 2D NMR NOESY spectrum of isomadecassoside (CD_3OD)

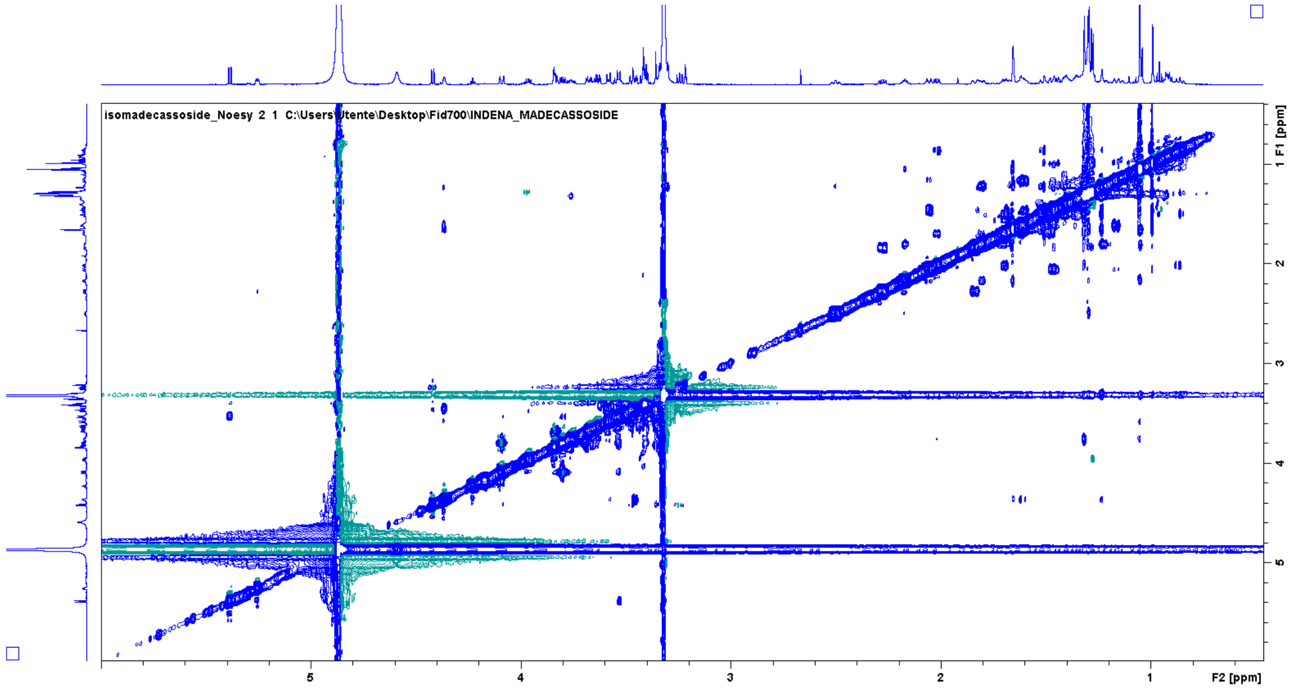

Supplement: Supplementary file 1 [file biomolecules-11-00494-s001.pdf]
